# Scarlet Fever

**Published:** 1898-09

**Authors:** H. B. Hill

**Affiliations:** Austin, Texas


For the Texas Medical Journal

### Scarlet Fever.

BY H. B. HILL, M. D., AUSTIN, TEXAS.

[Read before Austin District Medical Society.]

The object of this paper is to furnish a pretext to bring this subject before this society for discussion.

Scarlet fever is an acute infectious disease, classed among the exanthematica, and is accompanied by an affection of the throat, and usually also by albuminuria, as well as numerous complications, some of which will be referred to later.

It is caused by a peculiar poison, probably of bacterial origin, but, so far as I have been able to learn, its peculiar microbe has not been definitely determined yet. Whatever may be the nature of this poison, it retains its vitality for a long period of time. The period of contagion has been placed at various lengths; some authorities give from the first appearance of the rash to the date when all roughness of the heels and ankles has disappeared; a time varying from forty to eighty days. Under favorable circumstances clothing will remain contagious for a much longer period. Dr. Brooke, of United States army, reports a case contracted from infected clothing that had been packed in a trunk for one year. On the other hand, I had one case that was evidently caused by exposure to another case before the rash had appeared.

The poison is transmitted by a great variety of methods. The slighest contact is sufficient to communicate the disease. It may be conveyed by both the sick that have it and the well who have been with it; by articles of clothing, food and drink, by letters through the mails. Milk, which has a highly absorbent power, having frequently been the source of distribution.

There is a great difference of susceptibility, it being readily contracted by some individuals, while others, perhaps of the same family, escape. The age of the person has considerable influence; between two and three years being the most frequent period at which it is contracted. Infants under six months rarely ever have it, and the tendency to contract it being much diminished after puberty. Many persons who are brought in close contact with it, experience sore throat without having other symptoms. It is a well known fact that there is a remarkable prevalence of sore throat during an epidemic of scarlet fever. It seldom recurs in the same individual.

I will notice the pathological anatomy in connection with the symptoms.

There have been numerous forms described, but I will mention the symptoms of the more common forms only, and make a slight reference to rarer manifestations.

These are (1) the mild, (2) the anginose, and (3) the malignant. That is, (1) a scarlet fever without a throat affection, (2) one in which the throat is usually severely affected, and (3) the malignant type, which manifests itself in several ways. The onset is sudden and severe, usually with a more or less pronounced chill; the fever during the stage of invasion is often higher during this period than the corresponding period of the other eruptive fevers. Children often have convulsions during this stage or at the appearance of the eruption. Headache, vomiting, muscular pains, sometimes diarrhœa. Soreness of the throat may be mentioned as a symptom occurring during this stage, which usually is about twenty-four hours, but may be several days. There is a decided redness of the fauces several hours before the eruptive stage is reached. The eruption at first appears as minute specks which soon unite to form distinct patches which vary in size, and in many cases these patches unite in a general efflorescence, usually however retaining points of a more deeply red color which distinguish it from the appearance of erysipelas. It commonly makes its appearance on the body and limbs before it appears on the face, but to this rule there are numerous exceptions. In a few hours it spreads over the entire body, but in some severe cases it does not reach the maximum for several days. In these cases the eruption is often confluent, losing the dividing lines of normal skin between the patches, that are retained in the milder cases.

The tongue is at first thickly coated; peels off about the fourth day, leaving the "strawberry tongue," which is peculiar to scarletina, and by some authorities considered pathognomic. The urine at this time is scanty, highly colored, and contains more or less blood and albumen. In a few hours from the time the eruption reaches its maximum it begins to fade, requiring from three to seven days to complete this stage.

There is usually a condition varying from mental obtusion to active delirium during the height of the eruption, which disappears as the fever lessens; the eruption fades, and there is general improvement.

This stage is then gradually merged into that of desquamation which is at first of a fine scaly character, but soon patches of considerable size are thrown off, and occasionally, if the eruption has been very intense, casts of the entire cuticle of a finger or even of the whole hand may be stripped off, even including the nails, some times. In some cases there are several successive exfoliations. This stage is usually accompanied by more or less itching.

The duration of this stage may apparently be only five or six days, usually ten or twelve, but close observation will often show there is still remaining some evidence of it as late as forty or fifty days, or even longer, and until it is completed, the latest authorities consider there is still danger of infection.

The anginose form, which is by far the most common, is characterized by the symptoms above enumerated, and in addition, has an affection of the tissues in and around the throat, varying from a slight pharyngitis to an inflammation involving the pharynx, tonsils, cervical glands and adjacent tissues, which sometimes results in suppuration, and occasionally even in gangrene. Frequently when the throat affection is severe the eruption is slight.

Reference has been made to the character of the urine. These changes are accompanied by an involvement of the kidneys, varying from a slight hyperemia to a severe nephritis, resulting frequently in albuminuria and general dropsy. These renal changes are characterized by the various symptoms of uremic poisoning which, however, I will not detail here.

The malignant form may appear to be a mild case at first, but suddenly develops symptoms of a profound toxæmia, or at the very first there may be such an intensity of the poison as to overwhelm the system in a day or two. Once I had a case of this character, that in about forty-eight hours after the initial attack went suddenly into a profound stupor, the temperature rose to 107½°, the pulse became imperceptible, the breath came in gasps with the characteristic "death rattle" in the throat. In a short time there was every evidence of impending dissolution; but after free administration of stimulants and the constant sponging of the body with cool water until the temperature was brought down several degrees, there was a gradual amelioration of the symptoms, and the case finally resulted in recovery.

Among the other forms of the disease that I may mention, is the recurrent, which is a repetition of the same process after convalesence was apparently well under way, but which is usually somewhat milder than the first attack.

Another form is the hemorrhagic, in which the eruption is of dark color, irregularly developed, and it is probable these cases are frequently associated with diphtheria, as the throat is much swollen and diphtheritic exudations occur and hemorrhages take place, and death usually occurs early in the case.

On the question of diagnosis I must be brief, although the importance is manifest from the list of diseases from which it must be differentiated. These are: Erysipelas, small-pox, chicken-pox, measles, and roseola, etc. The diagnosis must be made by a comparison of the clinical history of each disease, and the character of the eruption. The possibility of scarlet fever, measles and diphtheria being associated in the same individual should be kept in mind, as persons who have had one or more of these diseases might be led to expose themselves, thinking they were protected from one, while the existence of two or more was not suspected.

Owing to the numerous complications and the erratic course of this disease the prognosis is rendered very uncertain.

Different epidemics vary so much in severity, that it has affected the literature of scarlet fever. Some writers have described it as a disease of so little importance as to scarcely merit a description, while others have found it among the most formidable. On account of the great fatality of epidemics in the community where I formerly practiced, that had occured previous to my advent there, scarlatina was more feared than yellow fever or small-pox, yet it is my good fortune to say that I have never lost a case. My brother-in-law, Dr. F. W. Flewellen treated about fifty cases in one epidemic, with the loss of only one case, toward the close of the epidemic In that case the poison was so intense as to cause death in forty-eight hours. The death rate is usually from ten to forty per cent in different epidemics.

In referring to the treatment of scarlet fever, I will confine myself principally to the more recent methods; presuming that the older methods are familiar to all present I will ignore them or only refer to them in a casual way.

Among recent writers there are three drugs that have been highly extolled by different authorities, and judging by the reports of cases treated by them, the results are very satisfactory, but I would here insert this caution, that is, that it is not safe to jump too hastily to a conclusion in regard to the treatment of scarlatina, as I am inclined to think the type of the epidemic is often to be credited with the results rather than the skill or want of skill in the practitioner, and also to the efficacy or want of efficacy in the drugs used. The three drugs referred to above are, acetate of ammonia, salicylic acid and chloral, the weight of testimony seeming to be in favor of the two last named. Vidal has recently recommended the use of acetate of ammonia in quantities of from a half drachm to a drachm and a half daily, with the result of rapidly reducing the temperature and establishing desquamation in about four days. It should be given early in the disease.

There is some very strong testimony as to the value of salicylic acid, both as a prophylactic and as a curative agent. De Rosa and Barken used it as a prophylactic in doses varying from 1½ to 4½ grains daily, with the following result: In sixty-six children belonging to families where there was scarlet fever, there occurred only three cases. Their conclusions are, that it is an absolute preventive if taken in time, and that it renders the disease mild if taken later.

Chakhovskoi recommends the salicylic acid treatment on the following grounds: One hundred and twenty-five malignant cases were treated, with only three deaths. He always employs the following formula:

℞ Acid Salicylic........................... grs. xv
Aqua distil. fervid..................... ℥ii
Syr. Aurant. Cor...................... ℥i

M. Sig. From a teaspoon to a tablespoon full every hour during the daytime, and every two hours during the night.

The solution is said to be perfect and palatable.

The following results are claimed for this treatment: In two or three days the temperature is reduced from 106° to 101½° or lower, reaching 98° about the tenth day of the treatment. To prevent relapses the remedy must be given every two hours for two or three days after deffervescence. Dr. Chakhovskoi states that this plan of treatment successfully prevents all complications (such as uræmia, dropsy, diphtheritic sore throats, adenitis, etc.), and even rapidly removes them when present.

The treatment by salicylic acid fails, according to his experience, when resorted to too late (after the fourth day), or when there are present certain severe chronic diseases, or serious congenital defects.

I will only say, by way of comment, that these results, if reliable, are sufficiently promising to make a trial desirable. I have had no personal experience with this method.

The chloral has had some very strong reasons advanced in its favor, but being potential for evil as well as for good, the prudent practitioner will be very cautious in its administration. First a laxative dose of calomel is given as soon as the patient is suspected or known to be taking the disease. Soon after, from one to five grains of chloral (according to the age of the patient) is given, to be repeated every two or three hours, or longer, throughout the attack; the object being to produce a quick, somnolent condition. The good effects claimed are, relief from restlessness, control of delirium, allaying the itching and burning of the skin, and the favorably influencing of a number of other distressing symptoms.

The author of the chloral treatment, Dr. J. C. Wilson, bases his theory on the following propositions:

1. The treatment of scarlet fever by chloral hydrate without the use of other drugs, has yielded satisfactory results.

2. The chief role of chloral in the treatment of scarlet fever is that of a sedative to the cerebral centers. It appears to antagonise certain exciting toxic principles formed within the organism during the course of the disease.

3. Chloral is useful also on account of its antiseptic properties (a) upon the throat; (b) upon the kidneys; (c) to a slight extent upon the fluids of the organism at large. It is necessary in this connection to bear in mind the difference between the germicide and the antiseptic influence of drugs. No amount of chloral compatible with the maintenance of life can act within the organism as a germicide. It is assumed that medicinal doses may tend to render the fluids of the body antiseptic; that is to say, may *tend* to impair to some extent, their fitness as culture media for pathogenic bacteria.

4. The elimination of chloral by the kidneys and its diuretic effect render it especially useful in the treatment of scarlet fever."

I have quoted this author's method thus fully, that you might have an opportunity of judging fairly its merits.

The line of treatment that I have usually adopted is, to put the patient upon a mixture of chlorate of potassa tincture of iron, etc., at the beginning. I then treat the general symptoms according to their indications. High temperature I reduce by sponging with cool water, to which some mild antiseptic is added, and in the interim the itching is allayed by applying some unctious substance containing a small quantity of carbolic acid. Depression and symptoms of heart failure I combat with alcoholic stimulants and digitalis, and also systematic feeding. In nephritis and albuminuria I have used dry cups over the loins, jaborandi, digitalis, saline diuretics, and drastic cathartics, when indicated. The adenitis would perhaps require additional treatment, similar to that indicated in inflammation from other causes. Obstetrical scarlatina in addition to the usual treatment for scarlet fever, would probably require similar treatment to that of puerperal septicæmia. Measures for prophylaxis should be of the strictest character, both in regard to the patients, the physicians, nurses, clothing, domicile, etc.

